# Surgical management of Marjolin’s ulcer secondary to chronic burn scar: a case report

**DOI:** 10.1093/jscr/rjag1009

**Published:** 2026-10-31

**Authors:** Yu Zhang, Jia Wang, Shengyuan Jin

**Affiliations:** Department of Burns and Plastic Surgery, The Affiliated Hospital of Yanbian University (Yanbian Hospital), No. 1327 Juzi Street, Yanji, Jilin 133000, China; Department of Gastroenterology, The Affiliated Hospital of Yanbian University (Yanbian Hospital), No. 1327 Juzi Street, Yanji, Jilin 133000, China; Department of Burns and Plastic Surgery, The Affiliated Hospital of Yanbian University (Yanbian Hospital), No. 1327 Juzi Street, Yanji, Jilin 133000, China

**Keywords:** burn scar carcinoma, Marjolin’s ulcer, squamous cell carcinoma, popliteal fossa

## Abstract

Marjolin’s ulcer is a rare malignancy originating from chronically injured skin with a prolonged latency period. We report a 71-year-old male with a 34-year history of a chronic burn scar ulcer in the left popliteal fossa that underwent malignant transformation. Histopathology confirmed moderately-to-well-differentiated squamous cell carcinoma. His comorbidities included stage 4 chronic kidney disease and hypertension. Following multidisciplinary assessment, wide local excision combined with punch skin grafting was performed. The grafts took well, and no recurrence was noted at short-term follow-up. Chronic post-burn scar ulcers require long-term surveillance, early biopsy, and individualized surgical planning, especially in patients with multiple comorbidities.

## Introduction

First described by Jean-Nicolas Marjolin in 1828, Marjolin’s ulcer is a rare and aggressive cutaneous malignancy that arises from chronic scars or non-healing wounds, most commonly following burns [1]. It predominantly affects males, and well-differentiated squamous cell carcinoma (SCC) is the most common histological subtype [2]. It is often diagnosed at an advanced stage and associated with a high mortality rate [3]. Heightened awareness of malignant transformation, prompt biopsy, and standardized treatment are therefore essential. We report a case of moderately-to-well-differentiated SCC arising from a 34-year-old burn scar ulcer in the left popliteal fossa in a patient with stage 4 chronic kidney disease, and discuss the diagnostic considerations, surgical strategy, and perioperative management.

## Case report

A 71-year-old male presented with a 34-year history of a chronic left popliteal fossa ulcer and a 2-month history of a progressively enlarging mass. Thirty-four years earlier, he sustained extensive gas-explosion burns and underwent skin grafting. A scar contracture subsequently developed in the left popliteal fossa, leading to recurrent ulceration; the wound was self-managed with topical povidone-iodine without formal care. Two months before admission, an exophytic mass appeared, and outpatient biopsy confirmed SCC. His history included poorly controlled hypertension (>10 years) and elevated serum creatinine (6 years), neither previously evaluated. Examination revealed limited left knee motion and a 5 × 4 × 3 cm cauliflower-like mass on the medial popliteal fossa, with necrotic crust and granulation tissue. The ulcer base measured 10 × 6 cm, with erythematous, friable margins, purulent exudate, foul odour, and contact bleeding (Fig. 1). No inguinal lymphadenopathy was palpable.

**Figure 1:**
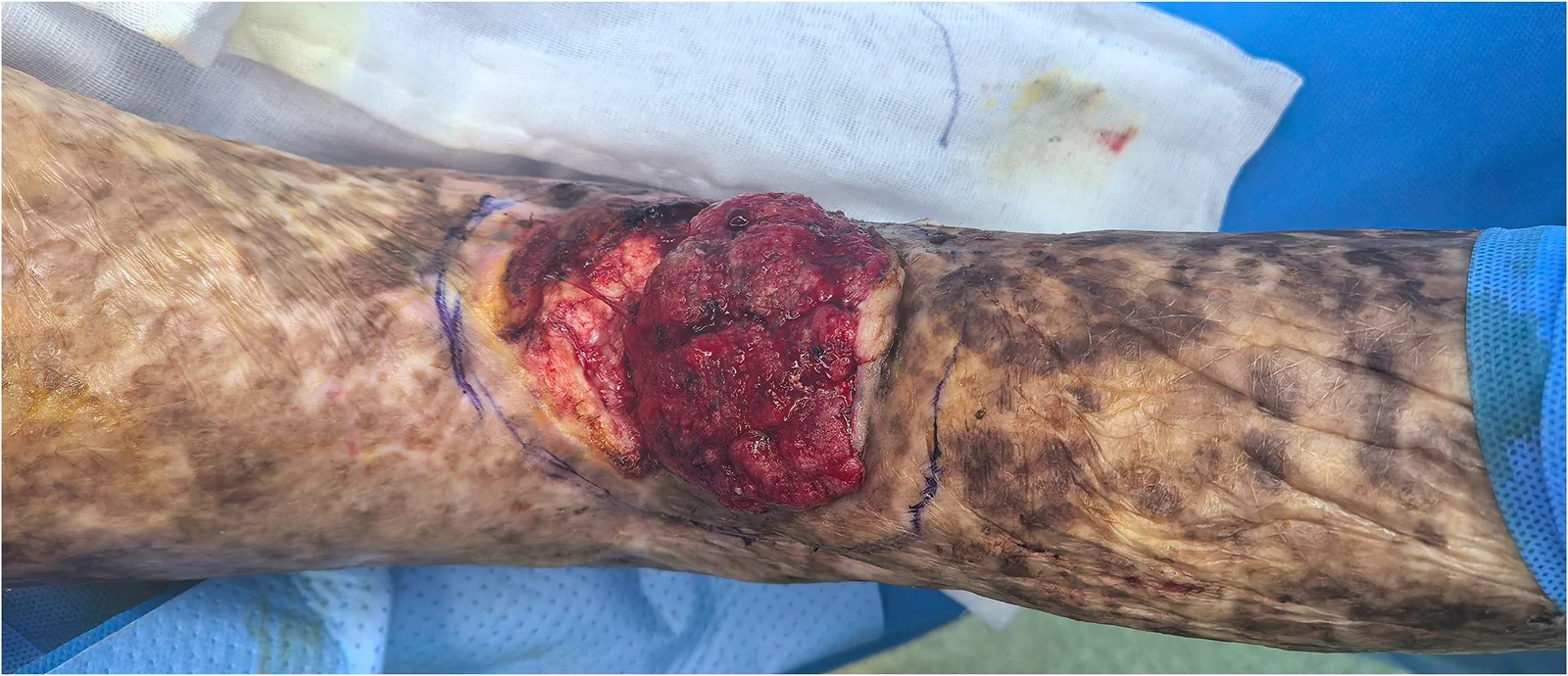
Preoperative clinical photograph of the left popliteal fossa. A cauliflower-like exophytic mass measuring 5 × 4 × 3 cm can be observed on the medial aspect of the popliteal fossa.

Laboratory tests showed haemoglobin 89 g/L, serum creatinine 221 μmol/L (estimated glomerular filtration rate 24.93 mL/min), and blood pressure 180/81 mmHg. Wound culture grew *Proteus mirabilis* susceptible to ceftazidime. Non-contrast computed tomography (CT) of the left knee revealed a 6.21 × 3.6 cm irregular soft-tissue mass in the popliteal region, without bone destruction or joint effusion. Echocardiography demonstrated left ventricular wall thickening and mild pulmonary hypertension. Intraoperative frozen sections showed moderately-to-well-differentiated SCC with clear peripheral and deep margins. Final histopathology revealed infiltrative moderately-to-well-differentiated SCC with subcutaneous abscess formation, no perineural or lymphovascular invasion, and an immunoprofile of p16(−), p53(+), Ki-67 70% (Fig. 2).

**Figure 2:**
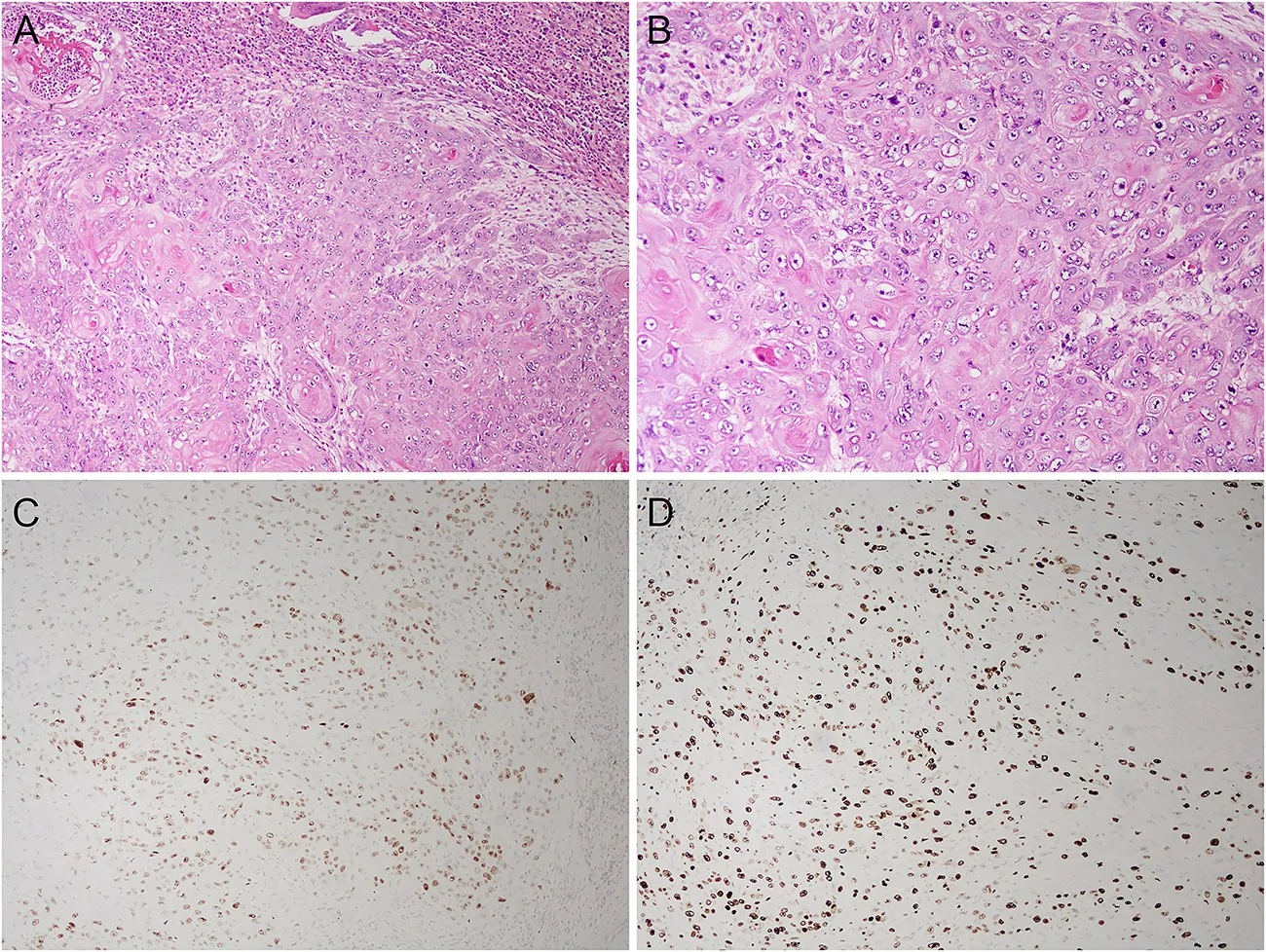
Histopathological and immunohistochemical features of the tumour. (A) Invasive squamous cell carcinoma with abundant keratin pearls and marked cellular pleomorphism (haematoxylin and eosin [H&E] stain, ×100). (B) Prominent nuclear atypia of tumour cells (H&E, ×200). (C) Positive nuclear p53 expression in tumour cells (p53 immunohistochemistry, ×100). (D) Ki-67 proliferative index of 70% in tumour cells (Ki-67 immunohistochemistry, ×100).

Intravenous ceftazidime was initiated. Multidisciplinary consultation (cardiology, nephrology, anaesthesiology) optimized blood pressure and surgical fitness. Under general anaesthesia, wide local excision with a 1.5-cm margin created a 14 × 9 cm defect extending to deep fascia (Fig. 3); frozen sections confirmed clear margins. Given extensive post-burn scarring and scarce donor sites, a split-thickness graft from the left buttock was fenestrated into punch grafts to resurface the defect. The donor site received allograft, and the limb was cast-immobilized. Blood loss was 100 mL; 1.5 units of leucodepleted packed red cells were transfused for renal anaemia. The patient tolerated the procedure well.

**Figure 3:**
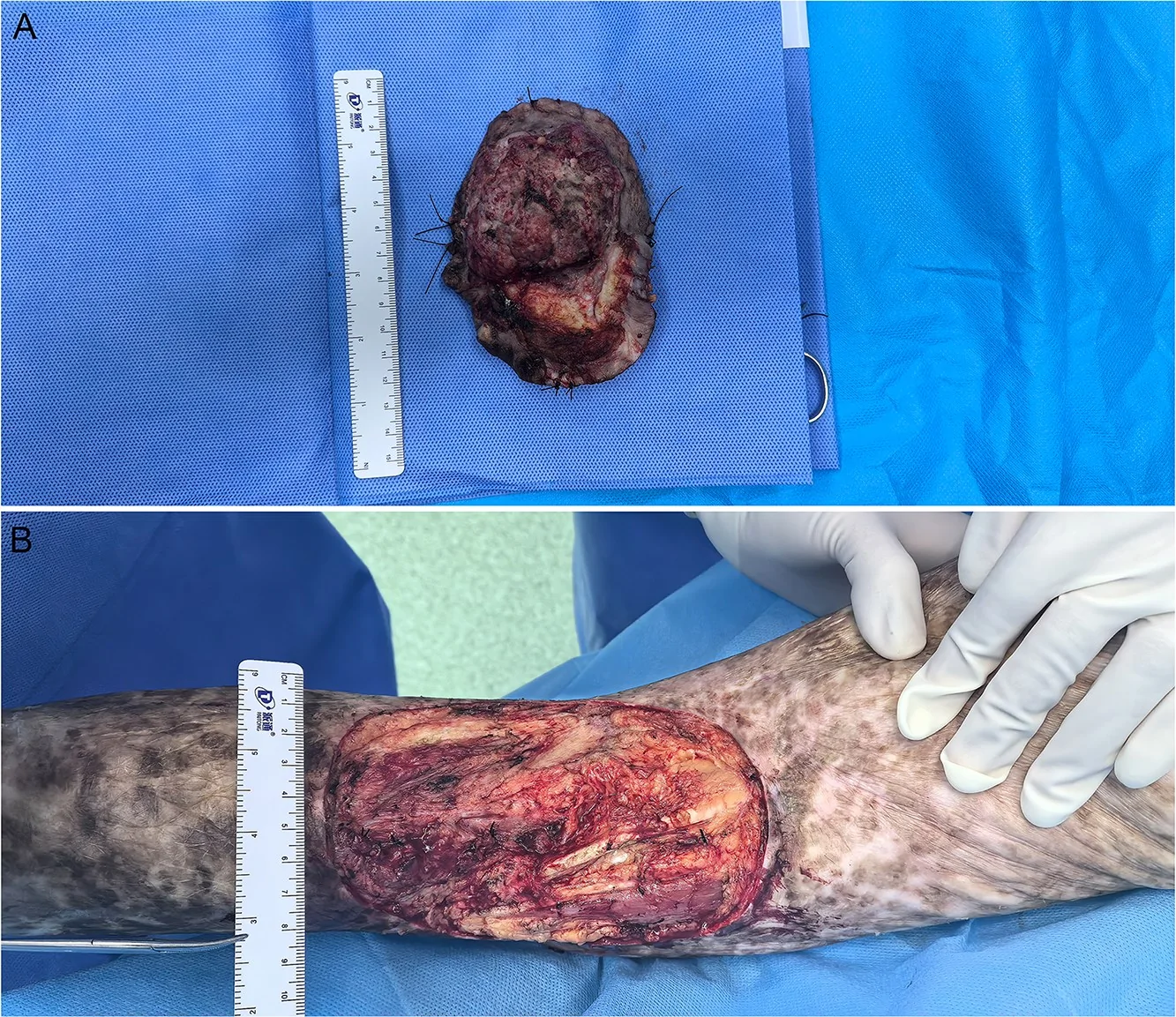
Gross specimen and intraoperative wound of the popliteal fossa lesion. (A) En bloc resected specimen with a ruler for scale. (B) Intraoperative view after wide local excision, showing the exposed deep fascial wound bed.

The cast was removed on postoperative day 7. Most grafts had taken well, with no haematoma or infection, and supportive care was continued. The patient was instructed in gentle active knee range-of-motion exercises and advised to avoid prolonged immobilization, without formal physiotherapy involvement. At the 2-month follow-up, the grafts were fully viable and had almost completely covered the wound (Fig. 4). The patient reported improved knee mobility, which was consistent with clinical observation. No local recurrence was observed, and inguinal lymph node ultrasound was unremarkable. The patient was satisfied and agreed to long-term follow-up.

**Figure 4:**
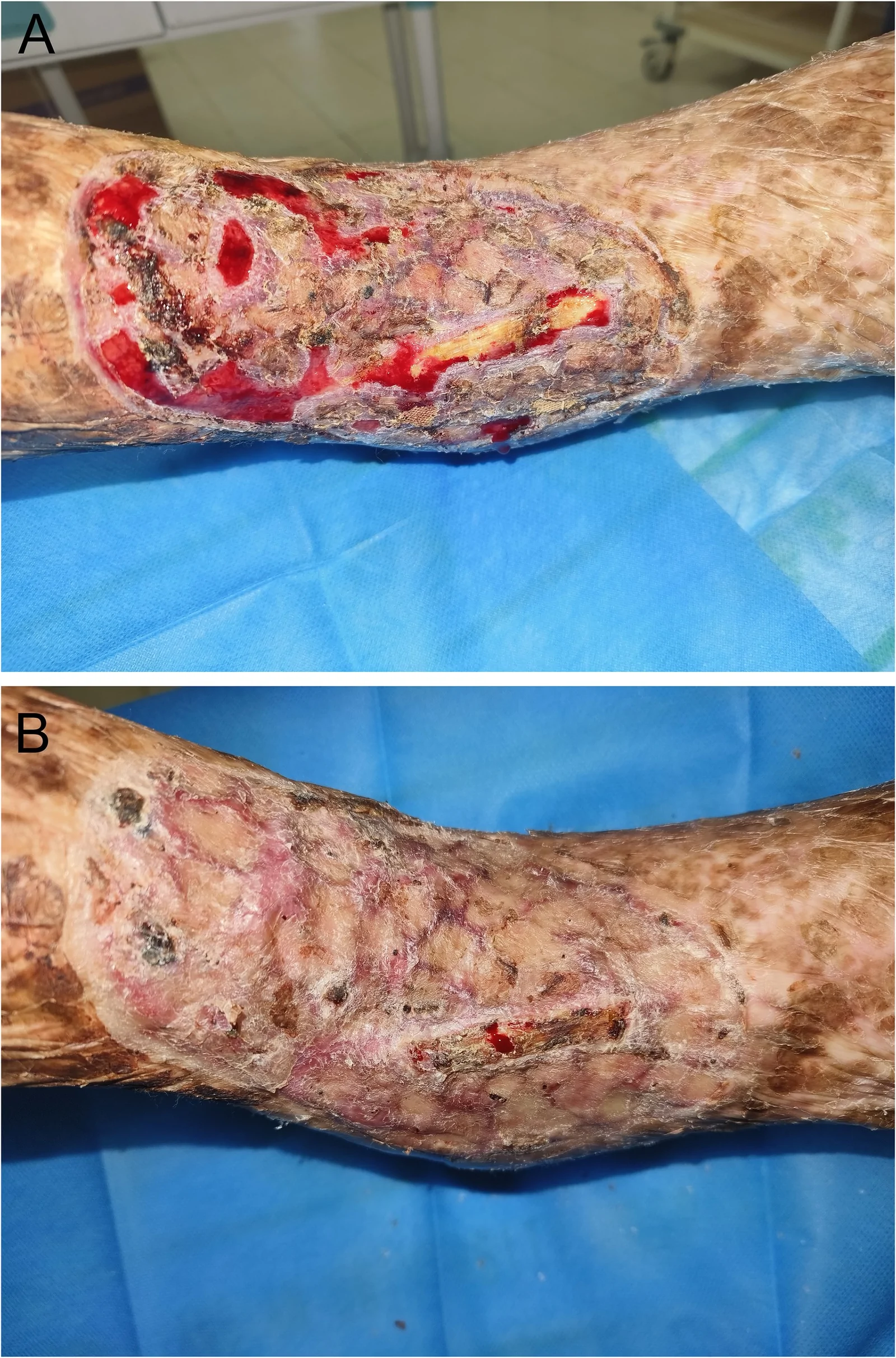
Postoperative wound healing progression. (A) Wound appearance at 1 month postoperatively, showing re-epithelialization spreading outwards from the punch skin graft sites, with some areas remaining unhealed. (B) Wound appearance at 2 months postoperatively, with nearly complete healing except for a small unhealed area over the tendon.

## Discussion

Marjolin’s ulcer develops in 0.77%–2% of burn scar patients, with a mean latency of 32 years [4]. The lower extremities are most commonly affected [5]. The popliteal fossa is subjected to repetitive traction and friction, predisposing to ulceration and malignant transformation [6]. In our patient, the ulcer remained untreated for 34 years, and care was sought only after a cauliflower-like mass appeared; by then the tumour measured 5 × 4 × 3 cm, underscoring the need for long-term scar surveillance and early biopsy [7].

Histopathology remains the gold standard for diagnosis. SCC accounts for approximately 86.72% of all Marjolin’s ulcers, with well-differentiated SCC being the predominant subtype [8]. Imaging is essential for evaluating the depth of invasion and bone involvement; osseous invasion has been reported in 27.8% of burn scar carcinomas [9]. In our patient, preoperative CT showed no bone destruction or joint effusion, creating favourable conditions for curative resection.

Wide local excision is the mainstay of treatment, used in 83.3% of cases in a 126-patient series [9], with intraoperative frozen section the reference standard for margin control [10]. We performed excision with a 1.5-cm margin and obtained real-time frozen-section confirmation of clear peripheral and deep margins, thereby ensuring oncological radicality and avoiding a second operation. For popliteal defects, flap reconstruction is theoretically preferred because it more effectively prevents postoperative scar contracture and preserves knee function [11]. However, flap reconstruction was considered unsuitable because the surrounding popliteal and thigh skin was heavily scarred, limiting reliable flap harvest, and the prolonged operative time and haemodynamic stress were poorly tolerated given the patient’s advanced age and stage 4 chronic kidney disease. A sheet split-thickness graft would require more donor skin than could be safely spared. We therefore performed punch skin grafting, a minimally invasive technique with low perioperative risk, minimal donor-site morbidity, and high healing rates in complex wounds [12], representing an individualized balance of oncological safety and functional outcome.

This case exhibited several favourable prognostic features—moderately-to-well-differentiated tumour, negative surgical margins, no bone invasion, and no palpable inguinal lymphadenopathy—consistent with node-negative, well-differentiated tumours <10 cm outside the head and neck, which carry a lower disease-specific mortality [8]. However, the Ki-67 index of 70% signals high proliferative activity and potentially greater local recurrence risk, warranting intensified surveillance. Follow-up must assess graft viability, local recurrence, regional nodes, and knee function.

In conclusion, chronic post-burn scar ulcers mandate long-term follow-up, and suspicious changes must undergo prompt biopsy. Wide local excision with clear margins remains the cornerstone of treatment. Wound reconstruction over joints should be individualized based on donor availability and functional needs. Elderly patients with comorbidities benefit from multidisciplinary care, and close postoperative surveillance is essential to detect early recurrence.

## Data Availability

All clinical data supporting the findings of this case report are included within the article. No additional publicly available datasets were generated or analysed during the current study.
